# Predictive value of serum uric acid-to-albumin ratio for diabetic kidney disease in patients with type 2 diabetes mellitus: a case-control study

**DOI:** 10.3389/fendo.2025.1577950

**Published:** 2025-10-07

**Authors:** Jing Du, Xiumei Xu, Ning Yuan, Xiaomei Zhang

**Affiliations:** Department of Endocrinology and Metabolism, Peking University International Hospital, Beijing, China

**Keywords:** type 2 diabetes mellitus, diabetic kidney disease, uric acid, albumin, inflammation

## Abstract

**Objective:**

The aim of this study was to investigate the predictive effects of the serum uric acid-to-albumin ratio (sUAR) on the onset of diabetic kidney disease (DKD) in patients with type 2 diabetes mellitus (T2DM).

**Methods:**

A case-control study was conducted involving 1809 patients with T2DM, including 486 cases with DKD and 1323 cases without DKD. Logistic regression and restricted cubic spline (RCS) analyses were used to analyze the relationship between the serum uric acid-to-albumin ratio (sUAR) and DKD. Receiver operating characteristic (ROC) curve was utilized to evaluate the predictive ability of the models for DKD. Decision curve analysis was performed to assess the clinical net benefit of the predictive models.

**Results:**

Multivariable logistic regression analysis revealed that sUAR was an independent risk factor for DKD (adjusted OR: 1.23, 95% CI: 1.16-1.30, *P* < 0.05). RCS analysis indicated a non-linear relationship between sUAR and DKD (*P* non-linear < 0.05). When sUAR exceeded 8.30, the risk of DKD gradually increased with higher sUAR. Additionally, combining sUAR with age, T2DM duration, glycated hemoglobin, systolic blood pressure, triglycerides, and high-density lipoprotein cholesterol significantly improved the predictive accuracy for DKD and the clinical net benefit.

**Conclusion:**

High sUAR levels were the independent risk factor of DKD. Moreover, incorporating sUAR with traditional influencing factors enhanced the predictive value for DKD.

## 1 Introduction

Diabetes mellitus (DM) has emerged as a critical global public health concern, with escalating prevalence rates. According to the latest data from the International Diabetes Federation (IDF), as of 2021, approximately 537 million people worldwide have been diagnosed with DM, and the number of patients will reach 783 million by 2045 (1). Among them, over 90% are classified as type 2 DM (T2DM) (2). Diabetic kidney disease (DKD) is one of the most serious microvascular complications of DM, affecting almost half of the individuals with T2DM (3–5). With the growing diabetic population, the burden of DKD also escalates. Currently, DKD has become the primary cause of chronic kidney disease (CKD) and end-stage renal disease (ESRD) (6). It substantially heightens the risk of cardiovascular events and all-cause mortality (7), leading to severe repercussions for individuals, families, and society. Moreover, it imposes a huge financial burden. Consequently, the proactive identification and management of risk factors linked to DKD are essential to prevent its onset and slow down its progression.

The pathogenesis of DKD involves a complex interplay of various mechanisms, including metabolic disturbances, hemodynamic alterations, and inflammatory response (3, 6). Serum uric acid (sUA) serves as a reliable biomarker for systemic inflammation and nutritional status (8). Elevated levels of sUA are associated with oxidative stress, inflammation, and endothelial dysfunction (9). sUA has been identified as an independent risk factor for CKD (10, 11). A recent Mendelian randomization analysis has further demonstrated a causal association between elevated sUA and the development of CKD (12). In patients with T2DM, previous studies have reported that sUA is closely related to the onset and progression of DKD (5, 13). Albumin (Alb), acting as a negative acute phase reactant, possesses anti-inflammatory and antioxidant properties (14). Studies have demonstrated that Alb is a protective factor in DKD (15) and that lower serum Alb concentration is associated with higher risk of ESRD (16). serum uric acid-to-albumin ratio (sUAR) is a novel composite biomarker with higher predictive value for inflammation and oxidative stress than sUA and Alb alone (17). However, there is a lack of data regarding the association between sUAR and DKD. Therefore, we conducted this study to explore the correlation between sUAR and DKD, and to evaluate its predictive value for DKD.

## 2 Material and methods

### 2.1 Ethics statement

The study received approval from the Ethics Committee of Peking University International Hospital [2022-KY-0030-03] and was conducted in adherence with the principles of Helsinki Declaration. In this study, due to its observational and retrospective nature and the absence of any potential harm or involvement of individual identifiable information, the requirement for written informed consent from the subjects was waived.

### 2.2 Study population

This was a case-control study. Patients admitted to the Department of Endocrinology at Peking University International Hospital from January 2017 to August 2021 were retrospectively reviewed. The inclusion criteria for the study were as follows: 1) adults aged 18 years or older; 2) patients diagnosed with T2DM according to the 1999 World Health Organization criteria; 3) patients with complete medical records. The exclusions criteria were as follows: 1) patients with gestational DM (GDM), type 1 DM (T1DM) or special types of DM; 2) patients with hematuria (including menstrual bleeding), other kidney diseases, or ESRD requiring dialysis; 3) patients with acute diabetic complications (such as ketoacidosis, hyperglycemic hyperosmolar state), acute infectious diseases, heart failure, liver diseases, autoimmune diseases, hematological diseases, and malignant tumors; 4) patients taking medications that could affect sUA and Alb levels. Ultimately, a total of 1,809 patients were included in this study.

### 2.3 Data collection

Demographic and clinical data, including age, sex, duration of type 2 diabetes mellitus (T2DM), medical history, and medication history, were collected. Blood pressure, weight, and height were measured and recorded by uniformly trained nurses. BMI (body mass index) was calculated as weight (kg)/height squared (m^2^).

Fasting peripheral venous blood samples were collected from all study subjects to detect the following indicators: glycated hemoglobin (HbA1c), fasting plasma glucose (FBG), high-density lipoprotein cholesterol (HDL-C), low-density lipoprotein cholesterol (LDL-C), total cholesterol (TC), triglycerides (TG), Alb, sUA and serum creatinine (sCr). The estimated glomerular filtration rate (eGFR) was calculated using the CKD-EPI equation (18). sUA and sCr were detected using the rate method. Serum Alb was detected using the bromocresol green method.

Morning urine samples were collected on three separate days to measure the urinary albumin/creatinine ratio (UACR), and the mean UACR (mUACR) was calculated. UACR was measured using the immune turbidimetry method.

### 2.4 Definitions and classification

The diagnosis of DKD required the presence of at least one of the following criteria, while excluding other causes of CKD: 1) In the absence of interfering factors, at least two out of three tests within 3–6 months showing UACR ≥ 30 mg/g. 2) eGFR < 60 ml/min/1.73 m² persisting for more than 3 months. 3) kidney biopsy demonstrating pathological changes consistent with DKD (19). According to the above diagnostic criteria, participants were divided into DKD and non-DKD group.

All patients were divided into 3 groups based on the tertiles of sUAR. The division criteria were as follows: tertile 1: sUAR < 7.41, tertile 2: 7.41≤ sUAR<9.40, tertile 3: sUAR ≥ 9.40.

### 2.5 Statistical analysis

Data analysis was performed using SPSS 24.0 and R 4.3.1 software. The normality of continuous data was assessed using the Shapiro-Wilk test. Continuous variables with normal distribution were presented as mean ± standard deviation, t-test was used for comparison between two groups, one-way analysis of variance was used for comparison among multiple groups, followed by *post-hoc* comparisons using the Bonferroni method (assuming homogeneous variance) or the Welch test (assuming heterogeneous variance); Continuous data with non-normal distribution were expressed as median and interquartile intervals, Mann-Whitney U test was used for comparison between two groups, Kruskal-Wallis H test was employed for comparisons among multiple groups, followed by pairwise comparisons using a rank test. Counting data were presented as frequency and percentage, chi-square test was used to compare between groups and linear-by-linear association chi-square test was used for trend test.

Univariate logistic regression and multivariate logistic regression were used to analyze the traditional risk factors of DKD. Logistic regression and restricted cubic spline (RCS) were used to analyze the correlation between sUAR and DKD. The Cochran-Armitage trend test was performed to analyze the effects of different degrees of sUAR on DKD.

A baseline model for DKD based on traditional risk factors was established. Furthermore, a nomogram model was developed by integrating sUAR with these traditional risk factors. This model was internally validated in cohorts using the bootstrap validation method by 1000 re-samplings. The predictive accuracy of the models was assessed using the receiver operating characteristic (ROC) curve and the area under the ROC curve (AUC). Net Reclassification Improvement (NRI) and Integrated Discrimination Improvement (IDI) were calculated to compare the incremental predictive performance of different models. The clinical utility of the models was evaluated using decision curve analysis (DCA). *P* < 0.05 was considered statistically significant.

## 3 Results

### 3.1 Comparison of characteristics between non-DKD group and DKD group

Among 1,809 patients with T2DM, 486 patients were complicated with DKD, yielding a prevalence of 26.9%. Compared with the non-DKD group, patients in the DKD group were older, and had a longer duration of diabetes. They also exhibited significantly higher levels of BMI, SBP, DBP, HbA1c, FPG, TG, mUACR, sUA and sUAR (*P* < 0.05). Conversely, levels of HDL-C, eGFR and Alb in patients with DKD were lower than those in patients with non-DKD (*P* < 0.05). There were no significant differences in the proportion of females and the levels of TC, and LDL-C between the two groups (*P >*0.05) (**Table 1**).

**Table 1:** Comparison of characteristics between non-DKD group and DKD group.

Variables	Total (N = 1809)	Non-DKD Group (n=1323)	DKD Group (n=486)	*P*
Age (years)	56.15 ± 13.62	54.89 ± 13.15	59.57 ± 14.29	<0.001
Female	682 (37.7%)	496 (37.5%)	186(38.3%)	0.761
T2DM Duration (years)	10.00 (5.00, 15.00)	9.00 (4.00, 14.00)	13.00 (8.00, 20.00)	<0.001
BMI (kg/m2)	25.89 ± 3.78	25.78 ± 3.70	26.20 ± 3.97	0.043
SBP (mmHg)	132.57 ± 17.44	130.42 ± 16.46	138.40 ± 18.67	<0.001
DBP (mmHg)	79.33 ± 11.05	79.02 ± 10.67	80.18 ± 11.98	0.046
HbA1c (%)	8.79 ± 1.99	8.66 ± 2.00	9.13 ± 1.93	<0.001
FPG (mmol/L)	9.13 ± 4.35	8.90 ± 4.24	9.77 ± 4.60	<0.001
TC (mmol/L)	4.34 ± 1.16	4.33 ± 1.08	4.38 ± 1.36	0.471
TG (mmol/L)	2.12 ± 1.95	2.04 ± 1.94	2.35 ± 1.97	0.003
HDL-C(mmol/L)	1.01 ± 0.28	1.03 ± 0.28	0.98 ± 0.28	0.002
LDL-C(mmol/L)	2.54 ± 0.92	2.56 ± 0.87	2.49 ± 1.03	0.178
eGFR (ml/min/1.73m2)	99.13 (87.92, 109.53)	100.68 (92.19, 110.86)	90.25 (67.95, 104.52)	<0.001
mUACR (mg/g)	10.36 (5.20, 31.12)	7.15 (4.24, 12.36)	72.25 (42.50, 206.88)	<0.001
sUA (umol/L)	345.07 ± 95.97	332.98 ± 86.52	377.98 ± 111.58	<0.001
Alb (g/L)	40.82 ± 3.70	40.96 ± 3.52	40.44 ± 4.12	0.014
sUAR	8.30 (6.92, 10.00)	8.02 (6.74, 9.57)	9.18 (7.57, 10.96)	<0.001

DKD, diabetic kidney disease; T2DM, type 2 diabetes mellitus; BMI, body mass index; SBP, systolic blood pressure; DBP, diastolic blood pressure; HbA1c, glycated hemoglobin; FPG, fasting plasma glucose; TC, total cholesterol; TG, triglycerides; HDL-C, high-density lipoprotein cholesterol; LDL-C, low-density lipoprotein cholesterol; eGFR, estimated glomerular filtration rate; mUACR, mean urinary albumin/creatinine ratio; sUA, serum uric acid; Alb, albumin; sUAR, serum uric acid-to-albumin ratio.

### 3.2 Comparison of characteristics across sUAR tertiles

**Figure 1:**
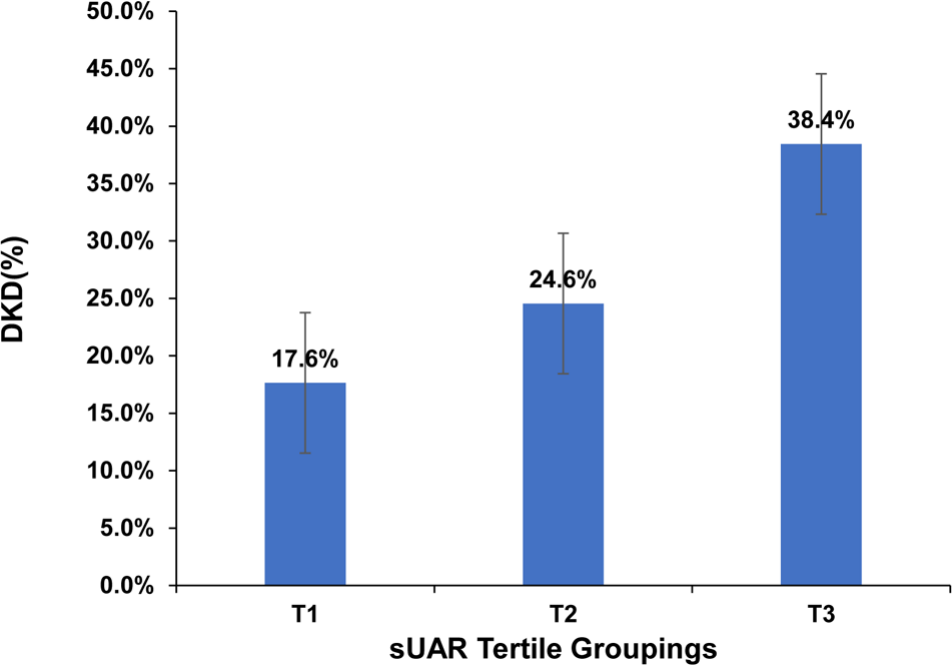
Prevalence of DKD across sUAR tertile groupings. DKD, diabetic kidney disease; sUAR, serum uric acid-to-albumin ratio.

Based on sUAR levels, we divided the patients with T2DM into 3 groups. The prevalence of DKD in tertile 1, tertile 2, and tertile 3 groups was 17.6%, 24.6%, and 38.4%, respectively. The result of linear-by-linear association chi-square test indicated that the incidence of DKD increased significantly with higher sUAR levels (*P*_trend_ < 0.05) (**Figure 1**). As shown in **Table 2**, compared to the other two groups, patients in tertile 1 group had the highest mean age and HbA1c, and the lowest levels of TG and mUACR (*P* < 0.05). Patients in tertile 3 group had the highest levels of BMI, DBP and sUA and the lowest levels of HDL-C, eGFR and Alb (*P* < 0.05). Additionally, the proportion of male patients was the highest in tertile 3 group (*P* < 0.05). Comparison with the tertile 1 group, patients in tertile 3 group had higher SBP levels and lower FPG levels (*P* < 0.05). Significant difference was not found in diabetic duration, TC and LDL-C among the three groups (*P* > 0.05).

**Table 2:** Comparison of characteristics across sUAR tertile groupings.

Variables	Tertile 1 (n=601)	Tertile 2 (n=607)	Tertile 3 (n=601)	*p*	Comparison between groups
Age (years)	57.44 ± 12.40	55.62 ± 13.33	55.39 ± 14.95	0.016	1>2, 1>3
Female	296(49.6%)	209(34.4%)	177(29.5%)	<0.001	1>2>3
T2DM Duration (years)	10.00(5.00, 15.00)	10.00(5.00, 15.00)	10.00(4.00, 17.00)	0.476	
BMI (kg/m2)	24.79 ± 3.47	25.88 ± 3.46	27.02 ± 4.06	<0.001	3>2>1
SBP (mmHg)	131.12 ± 17.60	132.50 ± 17.09	134.08 ± 17.52	0.013	3>1
DBP (mmHg)	78.74 ± 10.76	78.69 ± 10.47	80.57 ± 11.78	0.003	3>1, 3>2
HbA1c (%)	9.13 ± 2.13	8.68 ± 1.87	8.54 ± 1.93	<0.001	1>2, 1>3
FPG (mmol/L)	9.44 ± 3.67	9.14 ± 4.31	8.81 ± 4.97	0.047	1>3
TC (mmol/L)	4.33 ± 1.12	4.32 ± 1.07	4.37 ± 1.29	0.780	
TG (mmol/L)	1.78 ± 1.87	2.17 ± 2.01	2.42 ± 1.91	<0.001	3>2>1
HDL-C(mmol/L)	1.08 ± 0.31	1.00 ± 0.26	0.96 ± 0.27	<0.001	1>2>3
LDL-C(mmol/L)	2.58 ± 0.93	2.52 ± 0.85	2.52 ± 0.97	0.443	
eGFR (ml/min/1.73m2)	100.67 (93.20, 109.16)	99.71(89.98, 109.99)	94.64 (76.62, 109.94)	<0.001	1>2>3
mUACR (mg/g)	9.43 (5.39, 20.59)	9.81 (4.88, 27.96)	13.07 (5.14, 52.70)	<0.001	3>1, 2>1
sUA (umol/L)	251.13 ± 54.23	340.30 ± 35.52	443.83 ± 70.06	<0.001	3>2>1
Alb (g/L)	41.75 ± 3.74	40.91 ± 3.42	39.81 ± 3.68	<0.001	1>2>3
DKD	106(17.6%)	149(24.6%)	231(38.4%)	<0.001	3>2>1

sUAR, serum uric acid-to-albumin ratio; T2DM, type 2 diabetes mellitus; BMI, body mass index; SBP, systolic blood pressure; DBP, diastolic blood pressure; HbA1c, glycated hemoglobin; FPG, fasting plasma glucose; TC, total cholesterol; TG, triglycerides; HDL-C, high-density lipoprotein cholesterol; LDL-C, low-density lipoprotein cholesterol; eGFR, estimated glomerular filtration rate; mUACR, mean urinary albumin/creatinine ratio; sUA, serum uric acid; Alb, albumin; DKD, diabetic kidney disease

### 3.3 Traditional influencing factors of DKD

**Figure 2:**
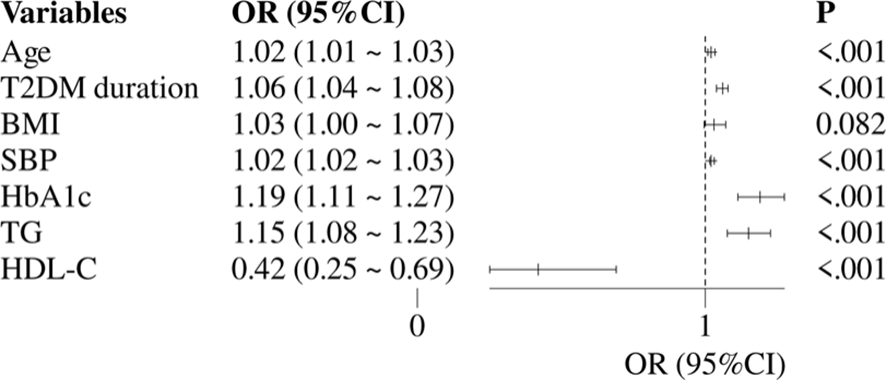
Multivariate logistic regression analysis of traditional risk factors for DKD. DKD, diabetic kidney disease; T2DM, type 2 diabetes mellitus; HbA1c, glycated hemoglobin; SBP, systolic blood pressure; HDL-C, high-density lipoprotein cholesterol; TG, triglycerides; OR, odds ratio; CI, confidence interval.

**Figure 2** constructed a multivariate logistic regression model with DKD as the dependent variable and with statistically significant variables from univariate regression analysis including age, T2DM duration, BMI, HbA1c, SBP, TG and HDL-C (excluding sUA, Alb, and sUAR) as independent variables. The results showed that DKD was independently associated with age (OR: 1.02, 95% CI: 1.01-1.03), T2DM duration (OR: 1.06, 95% CI: 1.04-1.08), SBP (OR: 1.02, 95% CI: 1.02-1.03), HbA1c (OR: 1.19, 95% CI: 1.11-1.27), TG (OR: 1.15, 95% CI: 1.08-1.23) and HDL-C (OR: 0.42, 95% CI: 0.25-0.69).

### 3.4 Association between sUAR and DKD

With DKD as the dependent variable and sUAR as the independent variable, univariate logistics regression analysis suggested that a high level of sUAR increased the risk of DKD (crude OR: 1.23, 95% CI: 1.18-1.29, *P* < 0.001). After adjusting for age, T2DM duration, HbA1c, SBP, TG and HDL-C, multivariate logistic regression analysis showed that sUAR was an independent risk factor for DKD (adjusted OR: 1.23, 95% CI: 1.16-1.30, *P* < 0.001). Specifically, the risk of DKD in tertile 2 and tertile 3 groups was 1.60 and 3.09 times higher, respectively, compared to the tertile 1 group. Additionally, the Cochran-Armitage trend test revealed an increasing trend in the risk of DKD with rising sUAR levels (*P*_trend_ < 0.05) (**Table 3**).

**Table 3:** Logistic regression analysis of sUAR and DKD.

Variables	Model 1		Model 2	
	Crude OR (95% CI)	*P*	Adjusted OR (95% CI)	*P*
sUA	1.01 (1.00, 1.01)	<0.001	1.01 (1.00, 1.01)	<0.001
Alb	0.96 (0.94, 0.99)	0.009	0.97 (0.93, 0.99)	0.038
sUAR	1.23 (1.18, 1.29)	<0.001	1.23 (1.16, 1.30)	<0.001
Tertile 1	Ref.		Ref.	
Tertile 2	1.52 (1.15, 2.01)	0.003	1.60 (1.14, 2.24)	0.007
Tertile 3	2.92 (2.23, 3.81)	<0.001	3.09 (2.19, 4.35)	<0.001
P _trend_	<0.001		<0.001	

Model 1: none (univariable); Model 2: adjusting for age, T2DM duration, SBP, HbA1c, TG and HDL-C; OR, odds ratio; CI, confidence interval.

DKD, diabetic kidney disease; sUAR, serum uric acid-to-albumin ratio; sUA, serum uric acid; Alb, albumin; T2DM, type 2 diabetes mellitus; SBP, systolic blood pressure; HbA1c, glycated hemoglobin; TG, triglycerides; HDL-C, high-density lipoprotein cholesterol.

**Figure 3:**
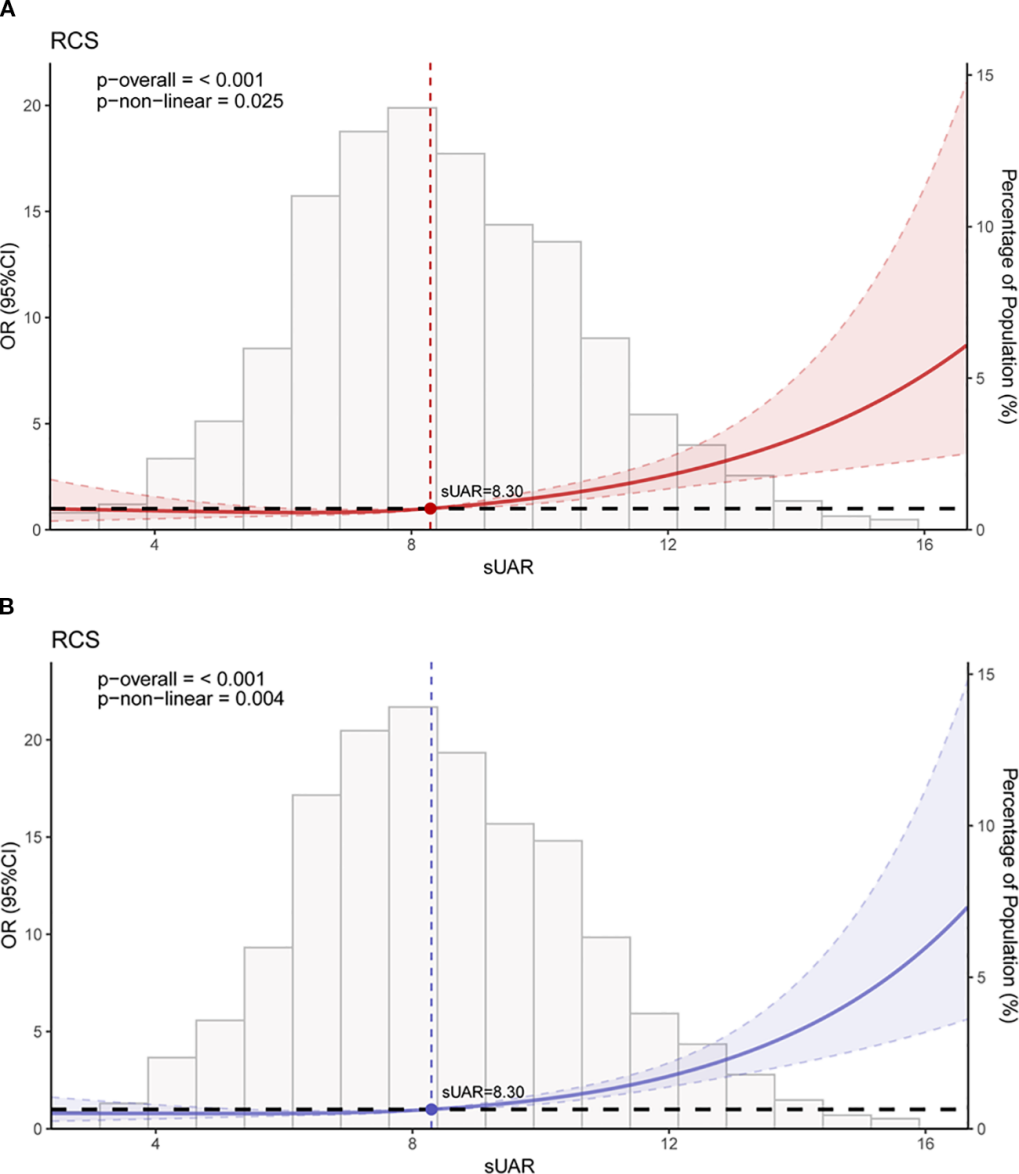
RCS curves of the association between sUAR and DKD. **(A)** unadjusted for any variables; **(B)** adjusted for age, T2DM duration, HbA1c, SBP, HDL-C, and TG. RCS, restricted cubic spline; sUAR, serum uric acid-to-albumin ratio; DKD, diabetic kidney disease; T2DM, type 2 diabetes mellitus; HbA1c, glycated hemoglobin; SBP, systolic blood pressure; HDL-C, high-density lipoprotein cholesterol; TG, triglycerides.

The RCS analysis results revealed a significant non-linear relationship between sUAR and the occurrence of DKD (*P* non-linear < 0.05). **Figure 3A** showed the unadjusted relationship, while **Figure 3B** was adjusted for age, T2DM duration, HbA1c, SBP, TG and HDL-C. Below a sUAR threshold of 8.30, the risk of DKD remained relatively constant. Nevertheless, exceeding this threshold led to a gradual escalation in the risk of DKD with increasing sUAR values.

### 3.5 Predictive model for the risk of DKD

**Figure 4:**
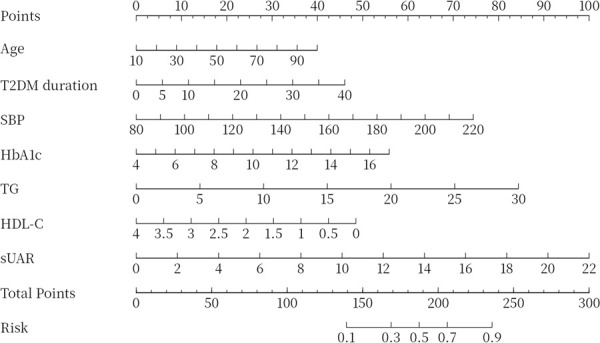
Nomogram for predicting DKD. DKD, diabetic kidney disease; T2DM, type 2 diabetes mellitus; HbA1c, glycated hemoglobin; SBP, systolic blood pressure; HDL-C, high-density lipoprotein cholesterol; TG, triglycerides; sUAR, serum uric acid-to-albumin ratio.

**Figure 5:**
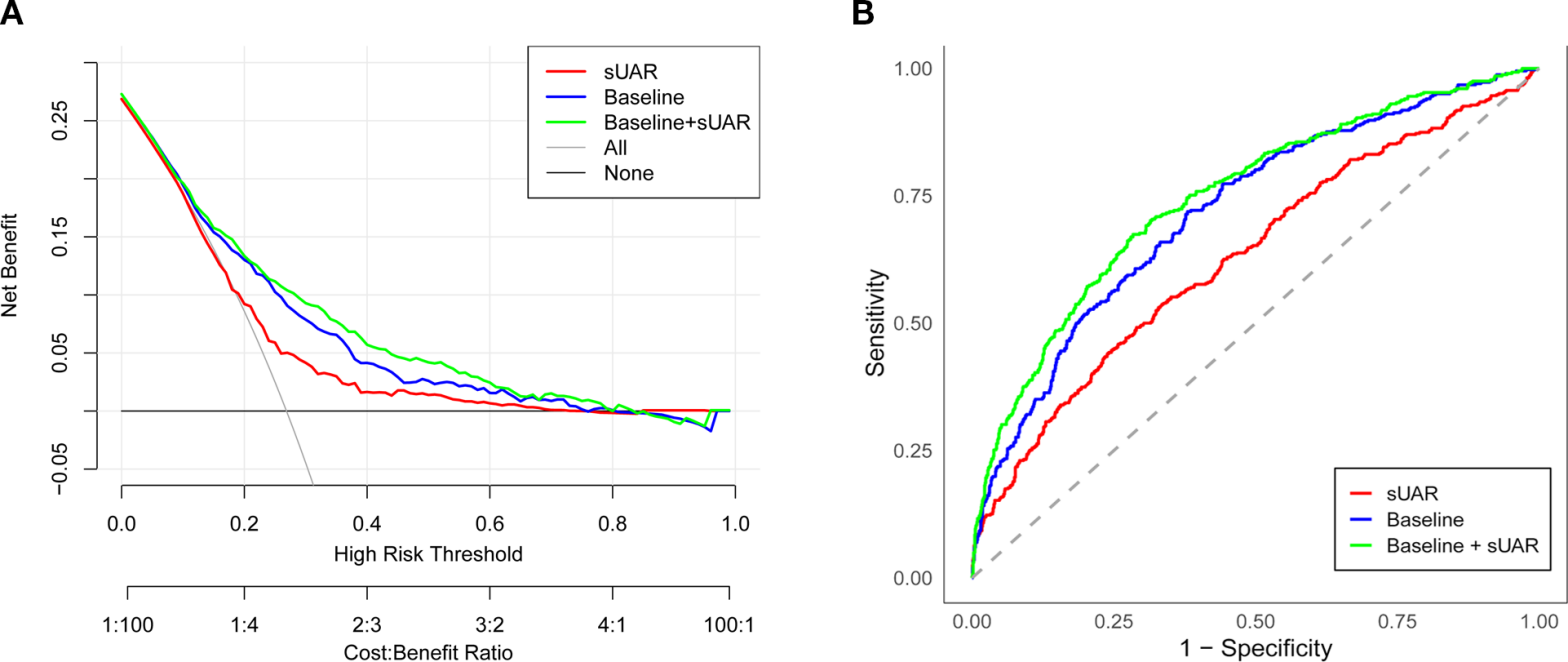
Evaluation of the prediction models for the risk of DKD. **(A)** ROC curve; **(B)** DCA curve. sUAR, serum uric acid-to-albumin ratio; DKD, diabetic kidney disease; ROC, receiver operating characteristic; DCA, decision curve analysis.

To evaluate the predictive performance of sUAR for DKD, a ROC curve analysis was performed, which yielded an AUC of 0.63 (95% CI: 0.60-0.66). The optimal threshold was 9.32, with the sensitivity and specificity of 67% and 54%, respectively. By integrating traditional influencing factors including age, T2DM duration, HbA1c, SBP, HDL-C, and TG, a baseline risk prediction model for DKD was constructed. This model demonstrated an AUC of 0.72 (95% CI: 0.69-0.75), with a sensitivity of 62% and a specificity of 72%. Furthermore, by combining sUAR with these traditional factors, an enhanced prediction model for DKD was developed based on nomogram (**Figure 4**). Internal validation revealed an improvement in prediction accuracy, with the AUC increasing to 0.75 (95% CI: 0.72-0.78). Sensitivity improved from 62% to 76%, and specificity increased from 72% to 87% (**Figure 5A**, **Table 4**). In **Table 4**, we also assessed these enhancements using continuous NRI and IDI. The continuous NRI was 0.37 (95% CI: 0.25-0.49, *P* < 0.001), and the IDI was 0.04 (95% CI: 0.03-0.05, *P* < 0.001).

**Table 4:** Discriminatory ability of the predictive models for DKD.

Models	AUC (95% CI)	Sensitivity	Specificity	Continuous NRI (95% CI)	P	IDI (95% CI)	P
sUAR model	0.63 (0.60, 0.66)	0.67	0.54				
Baseline model	0.72 (0.69, 0.75)	0.62	0.72	Ref.		Ref.	
Baseline +sUAR model	0.75 (0.72, 0.78)	0.76	0.87	0.37 (0.25, 0.49)	<0.001	0.04 (0.03, 0.05)	<0.001

Baseline model including age, T2DM duration, HbA1c, SBP, HDL-C, and TG.

DKD, diabetic kidney disease; sUAR, serum uric acid-to-albumin ratio; AUC, area under the curve; CI, confidence interval; NRI, net reclassification improvement; IDI, integrated discrimination improvement.

In addition, the results of DCA found that when the threshold probability was between 0.16 and 0.52, the clinical net benefit was ranked as baseline + sUAR model > baseline model > sUAR model (**Figure 5B**).

## 4 Discussion

DKD is a form of CKD that develops as a complication of DM. It is characterized by persistent increased urinary albumin excretion and/or decreased eGFR. Previous studies have reported that DKD affects 20%-50% of diabetic patients (20), with 30%-50% of cases attributed to T2DM (13). DKD has become a significant global public health issue due to its high prevalence, poor prognosis, and heavy economic burden. Given these challenges, it is crucial to identify and intervene in the risk factors associated with DKD at an early stage.

sUA is a metabolite of purine produced by xanthine oxidase (21, 22). Several studies have demonstrated that hyperuricemia is an independent risk of DKD (5, 13, 23). A meta-analysis involving 27,425 T2DM patients revealed that for every 1 mg/dL increase in sUA levels, the risk for DKD increased by 11% (RR = 1. 11, 95% CI: 1.05-1.17, *P* < 0.0001); and the result of subgroup analysis further demonstrated a linear relationship between sUA levels and the presence of albuminuria (*P* = 0.052) as well as eGFR decline *(P* < 0.0001) (13). Kim WJ, et al. found that sUA > 327 μ mol/L was significantly associated with an eGFR below 60 ml/min/1.73 m 2 in T2DM patients with renal function-preserving (HR = 2. 97, 95% CI: 1.15-7.71, *P* = 0.025). Furthermore, their study suggested that even when sUA levels were within the normal range, higher sUA levels were still associated with a decline of renal function (23). Another cross-sectional study indicated that T2DM patients with higher sUA levels have a higher risk of DKD progression (5). Previous studies have suggested that sUA-lowering therapy can reduce proteinuria (24, 25) and slow the progression of renal function deterioration (26). The mechanism of sUA affecting DKD has not been fully elucidated. High sUA level can activate the NOD-like receptor thermal protein domain-associated protein 3 (NLRP3) inflammasome, leading to cell damage and even death through endoplasmic reticulum stress, lysosome destruction, mitochondrial dysfunction, and the interaction between the Golgi apparatus and extracellular vesicles (27). In addition, hyperuricemia can stimulate the renin-angiotensin system, damaging vascular smooth muscle cells, causing renal artery lesions, and increasing intrarenal pressure, ultimately leading to microvascular injury (28). Another possible mechanism is that elevated sUA induces the production of inflammatory cytokines, including tumor necrosis factor-α, interleukin (IL)-1β, IL-6, and IL-17, which promote renal damage (29, 30). In our study, we found that sUA levels in DKD group were higher than those in non-DKD group (377.98 ± 111.58 vs. 332.98 ± 86.52, *P* < 0.05). High levels of sUA were associated with an increased risk of DKD (adjusted OR: 1.01, 95% CI: 1.00-1.01, *P* < 0.05).

Apart from being a major determinant of intravascular osmotic pressure, Alb also plays a crucial role in anti-inflammatory, anti-apoptotic, and antioxidant functions (14, 31, 32). A prospective study based on the UK Biobank has indicated that serum Alb levels were inversely associated with the incidences of diabetes and DKD (15). A study involving 188 T2DM patients with biopsy-proven DKD demonstrated a significant relationship between serum Alb and proteinuria, renal dysfunction, and glomerular lesions (31). Furthermore, lower serum Alb levels were associated with a higher risk of developing ESRD (16, 31). Moreover, some studies have found that decreased Alb levels increase the risk of all-cause mortality in patients with DKD (33). Our research findings indicated that the levels of Alb in the DKD group are lower than those in the non-DKD group (40.44 ± 4.12 vs. 40.96 ± 3.52, *P* < 0.05). Additionally, a decrease in Alb was associated with an increased risk of DKD (adjusted OR: 0.97, 95% CI: 0.93-0.99, *P* < 0.05).

Previous studies have shown that inflammation plays an important role in the occurrence and progression of DKD. As a novel inflammatory biomarker, sUAR combines the pro-oxidative effects of sUA with the antioxidant properties of Alb, potentially providing a more comprehensive reflection of the body’s inflammatory and oxidative stress status. Consequently, sUAR has garnered increasing attention in recent research. Yeter Hh, et al. reported a significant association between sUAR levels above 1.7 and the development of acute kidney injury (AKI) (34). Liao Y, et al. also demonstrated that elevated sUAR was significantly associated with an increased risk of AKI in patients undergoing isolated tricuspid valve surgery (35). Furthermore, Özgür Y, et al. identified that higher sUAR levels were linked to increased higher 30-day mortality rates in patients with AKI (31). Şaylık F, et al. and Zhang Y, et al. found that sUAR was an independent risk factor for post-angiographic kidney injury in patients with ST-segment elevation myocardial infarction who underwent percutaneous coronary intervention (36, 37). This study, for the first time, identified a correlation between sUAR and DKD. With increasing sUAR levels, a rising trend in the incidence rate of DKD was observed (*P*_trend_ < 0.05). After adjusting for age, T2DM duration, HbA1c, SBP, TG and HDL-C, multivariable logistic regression analysis showed that sUAR was an independent risk factor for DKD (adjusted OR: 1.23, 95% CI: 1.16-1.30, *P* < 0.001). RCS analysis indicated a non-linear relationship between sUAR and the occurrence of DKD (*P* non-linear < 0.05). Specifically, when sUAR exceeded 8.30, the risk of DKD occurrence gradually increased with higher sUAR values.

The results of this study also found that older age, longer T2DM duration, low HDL-C levels, and high levels of HbA1c, SBP, and TG were risk factors for DKD, consistent with previous studies (38, 39). Furthermore, we found that incorporating both sUAR and the traditional influencing factors of DKD into a multivariable prediction model can improve the predictive accuracy and clinical net benefit. In addition, we developed a visual nomogram that allowed both doctors and patients to more intuitively understand the prediction results for DKD. This model is especially beneficial for primary care hospitals that lack the capability to measure UACR or perform renal biopsies, as it enhances early screening for DKD.

There are several limitations to this study. First, this is a single-center study involving hospitalized patients, which may lead to some biases. Second, potential effects of antidiabetic drugs (e.g., insulin, sodium-glucose cotransporter-2 inhibitors) on sUA and Alb were not considered. Third, the diagnosis of DKD predominantly relied on elevated UACR and reduced eGFR, with renal biopsy performed in only a minor subset of cases.

## 5 Conclusion

In summary, this study revealed that high levels of sUAR were independent risk factors of DKD, and sUAR combined with traditional risk factors had a higher predictive value for DKD.

## Data Availability

The raw data supporting the conclusions of this article will be made available by the authors, without undue reservation.
